# Regulation of *miR-483-3p* by the O-linked *N*-acetylglucosamine transferase links chemosensitivity to glucose metabolism in liver cancer cells

**DOI:** 10.1038/oncsis.2017.35

**Published:** 2017-05-08

**Authors:** F Pepe, S Pagotto, S Soliman, C Rossi, P Lanuti, C Braconi, R Mariani-Costantini, R Visone, A Veronese

**Affiliations:** 1 Department of Medical, Oral and Biotechnological Sciences, G. d’Annunzio University, Chieti, Italy; 2 Unit of General Pathology, Aging Research Center and Translational Medicine (CeSI-MeT), G. d’Annunzio University, Chieti, Italy; 3 Department of Experimental and Clinical Sciences, G. D'Annunzio University, Chieti, Italy; 4 Department of Medicine and Aging Science, University G. d’Annunzio Chieti-Pescara, Chieti, Italy; 5 Division of Cancer Therapeutics, Institute of Cancer Research, London, UK

## Abstract

The *miR-483-3p* is upregulated in several tumors, including liver tumors, where it inhibits TP53-dependent apoptosis by targeting the pro-apoptotic gene *BBC3*/PUMA. The transcriptional regulation of the *miR-483-3p* could be driven by the β-catenin/USF1 complex, independently from its host gene *IGF2,* and we previously demonstrated that in HepG2 hepatoblastoma cells carrying wild-type *TP53* the upregulation of the *miR-483-3p* overcomes the antitumoral effects of the tumor-suppressor *miR-145-5p* by a mechanism involving cellular glucose availability. Here we demonstrate that in HepG2 cells, the molecular link between glucose concentration and *miR-483-3p* expression entails the O-linked N-acetylglucosamine (O-GlcNAc) transferase (OGT), which stabilizes the transcriptional complex at the *miR-483* promoter. HepG2 cells showed reduced *miR-483-3p* expression and increased susceptibility to 5-fluorouracil (5-FU)-induced apoptosis in presence of the inhibitor of glycolysis 2-deoxy-d-glucose (2-DG). However, *in vivo* experiments showed that HepG2 cells with higher *miR-483-3p* expression were selected during tumor progression regardless of 5-FU treatment. Furthermore, treatment with 2-DG alone did not significantly reduce HepG2 xenograft load in immunodeficient mice. In conclusion, we show that in HepG2 cells glucose uptake increases the expression of the oncogenic *miR-483-3p* through the OGT pathway. This suggests that depletion of the *miR-483-3p* may be a valuable therapeutic approach in liver cancer patients, but the use of inhibitors of glycolysis to achieve this purpose could accelerate the selection of resistant neoplastic cell clones.

## Introduction

Hepatocellular carcinoma (HCC) is the fifth most common form of cancer worldwide and the third cause of cancer-related deaths.^1, 2^ The current therapies are limited and often ineffective, thus there is a need to identify new druggable molecular targets for the development of novel therapeutics.

We have previously shown that *miR-483-3p* is overexpressed in HCCs that carry mutations in β-catenin pathway genes^3^ and in HCCs with wild-type *TP53,* as compared with those with mutated *TP53*.^4^ The miR is transcribed through the β-catenin (CTNNB1)/USF1 complex and targets the important downstream apoptotic factor of TP53, PUMA.^3, 5^ This gives to the miR a role in HCC tumorigenesis, based on the induction of resistance to apoptosis. Indeed, we have previously shown that in hepatoblastoma HepG2 cells, which carry mutated CTNNB1 and wild-type TP53, constant activation of the TP53/*miR-145-5p* signaling selects cells with high *miR-483-3p* expression that are resistant to apoptosis. Depletion of *miR-483-3p* leads to apoptosis of these cells. We also demonstrated that *miR-145-5p* influences the expression of *miR-483-3p* in HepG2 cells with stimulatory or inhibitory effects, depending on the availability of glucose in the culture media.^4^ The involvement of glucose metabolism in the regulation of *miR-483-3p* is supported by several studies that connect CTNNB1 and USF1 activity to cellular glucose metabolism,^6, 7, 8^ and by the fact that *miR-483-3p* maps at the *INS-IGF2* locus, which is involved in the insulin pathway.^9, 10^

Here we show that in HepG2 cells the expression of *miR-483-3p* is affected by the extracellular concentration of glucose. In fact both glucose starvation and treatment with the glucose-mimic 2-DG reduce *miR-483-3p* expression. We identify as a key factor of this regulation the O-linked β-*N*-acetylglucosamine transferase (O-GlcNAc transferase, OGT). OGT is responsible for O-linked β-*N*-Acetylglucosamine (O-GlcNAc) protein modifications using the activated derivative of glucose metabolism Uridine diphosphate *N*-acetylglucosamine or UDP-GlcNAc as substrate. Therefore, protein O-GlcNAcylation is considered a cellular nutrient sensor and a metabolic regulator.^11, 12^

On the basis of our previous study, which showed increased cell death susceptibility in HepG2 cells following depletion of the *miR-483-3p* after inhibition of glucose metabolism, we tested 2-DG as an adjuvant treatment, combined with 5-fluorouracil (5-FU), on a murine xenograft model with tumors induced by peritoneal injection of HepG2 cells.

## Results

### Glucose concentration affects *miR-483-3p* expression in HepG2 cell line

**Figure 1:**
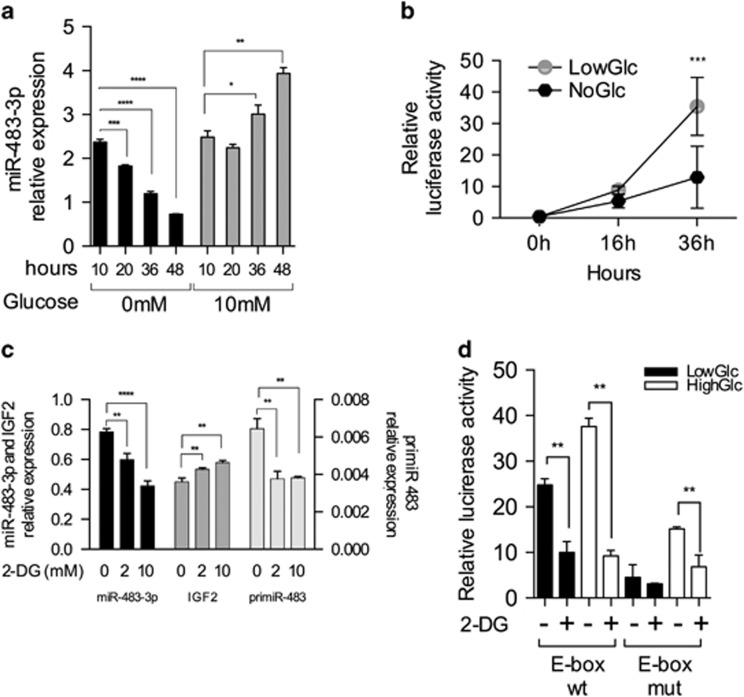
Glucose deprivation and 2-DG treatment reduce *miR-483-3p* expression in HepG2 cell lines. (**a**) *MiR-483-3p* relative expression by RT–qPCR in HepG2 cells cultured in Dulbecco’s modified Eagle’s medium (DMEM) media without glucose (black bars) or with glucose 10 mM (gray bars) for 10, 20, 36 and 48 h. (**b**) Relative luciferase activity of the *miR-483* promoter sequence containing the E-Box interacting with CTNNB1/USF1 complex, in HepG2 cells cultured in DMEM media without glucose (black circles) or with glucose 10 mM (gray circles) for 0, 16 and 36 h. (**c**) *MiR-483-3p*, *IGF2* (left *y* axis) and *primiR-483* (right *y* axis) relative expression by RT–qPCR in HepG2 cells treated with 2-DG at 2 and 10 mM for 48 h. *MiR-483-3p* expression was normalized on U44, whereas *IGF2* and *primiR-483* on ACTB. (**d**) Relative luciferase activity of the *miR-483* promoter sequence containing the E-Box interacting with CTNNB1/USF1 complex, in HepG2 cells treated with 2-DG 5 mM in low (black bars) or high (white bars) glucose condition (1 and 4.5 g/l, respectively) for 48 h. As control for the wild-type vector (wt) was used mutated vector (mut) for the region interacting with the CTNNB1/USF1 complex. The graphs represent the means of technical triplicates with the respective s.d. For statistical analysis, Student’s *t*-test was applied (*P<0.05; **P<0.01; ***P<0.001; ****P<0.0001).

*MiR-483-3p* is regulated by the transcriptional factors CTNNB1 and USF1, therefore, on the basis of our previous observations, we speculated that glucose deprivation could reduce the expression of this miRNA. To study the effect of glucose deprivation on *miR-483-3p* expression, we cultured HepG2 cells with either no-glucose or 10 mM glucose, and we collected cells at 10, 20, 36 and 48 h. We observed a gradual and significant reduction of *miR-483-3p* expression in cells cultured in no-glucose condition; on the contrary, *miR-483-3p* expression increased over time in the presence of glucose (Figure 1a).

To investigate if the CTNNB1/USF1 complex could have a direct role on the increase of *miR-483-3p* expression in response to glucose in HepG2 cells, we assayed the luciferase activity of a vector carrying the E-Box-responsive element to CTNNB1/USF1 complex.^3^ The luciferase values showed reduced activity in no-glucose relative to low glucose condition, indicating that the glucose-dependent *miR-483-3p* regulation could be transcriptionally controlled by CTNNB1/USFl (Figure 1b).

To strengthen these data, we treated HepG2 cells with the glucose antagonist 2-deoxy-d-glucose (2-DG) for 48 h. We observed a significant reduction of the levels of both the *miR-483-3p* and its precursor *pri miR-483* in a 2-DG concentration-dependent way. The host gene *IGF2* was not affected, suggesting that glucose deprivation regulated the transcription of *miR-483-3p* independently of IGF2 (Figure 1c).

To establish if 2-DG treatment affected *miR-483-3p* expression by altering the activity of the CTNNB1/USF1 complex, we measured the luciferase activity of the vectors carrying either wild-type or mutated CTNNB1/USF1-responsive elements in HepG2 cells. Cells were grown in low or high glucose media and treated or not with 2-DG. The assay showed that, upon 2-DG treatment, the promoter was less activated in both glucose conditions and the response to 2-DG was absent or much less evident using the vector harboring the mutant sequence (Figure 1d). Overall, these results indicate the involvement of the CTNNB1/USF1/E-box complex in the regulation of *miR-483-3p* expression.

**Figure 2:**
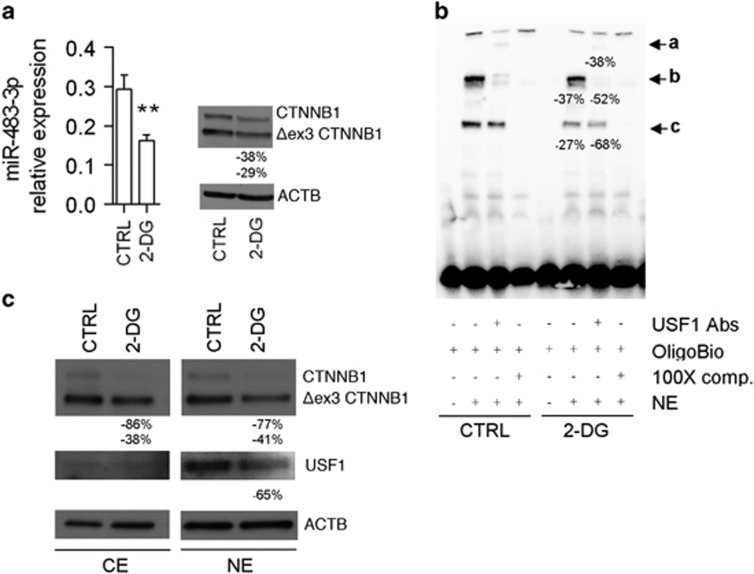
2-DG treatment affects *miR-483-3p* expression at transcriptional level. (**a**) *miR-483-3p* relative expression by RT–qPCR (left panel), and CTNNB1 (wild-type and mutated) protein relative expression by western blot (right panel), in HepG2 cells treated with 2-DG 20 mM for 2 weeks in high glucose. The graphs represent the means of technical triplicates with the respective s.d. For statistical analysis, Student’s *t*-test was applied (**P<0.01). The right panel shows the percentage variation with respect to the relative control by densitometry analysis. (**b**) Electrophoretic mobility shift assay (EMSA) of nuclear extract (NE) from HepG2 cells treated with 2-DG 5 mM using the *miR-483* E-box probe. The specific complexes are indicated with black arrows (b, c). Arrow a shows the supershift generated by USF1 complex. Densitometry analysis is shown. EMSA was performed once. (**c**) Protein relative expression of cytoplasmic (CE) and nuclear (NE) CTNNB1 and USF1 by western blot. Densitometry analysis is shown.

To further investigate this hypothesis, we treated HepG2 cells with 2-DG for 2 weeks and we observed a significant reduction of the levels of *miR-483-3p* and CTNNB1 (Figure 2a). Next, we evaluated the activity of the CTNNB1/USF1 complex on the *miR-483* promoter in HepG2 cells upon 2-DG treatment. The compound was able to reduce the interaction between the CTNNB1/USF1 complex and the probe, as demonstrated by the reduction of the specific bands ‘b’ (37%) and ‘c’ (–27%). The specificity of the complex was demonstrated by the supershift using the anti-USF1 antibody (band ‘a’) that showed a reduction of intensity of about 38% after treatment (Figure 2b). We also noted a reduction of the nuclear fraction of mutated CTNNB1 and USF1 in cells treated in the same conditions (Figure 2c). These findings suggest the 2-DG influences *miR-483-3p* expression by reducing both the amount of the CTNNB1/USF1 complex and its affinity for the E-box element upstream the *miR-483* gene.

### OGT activity regulates *miR-483-3p* expression acting at the transcriptional level

**Figure 3:**
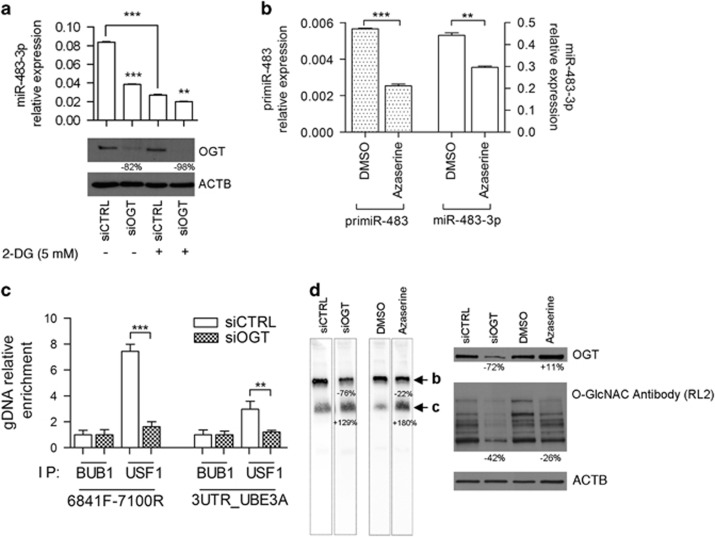
OGT activity regulates *miR-483-3p* expression. (**a**) *MiR-483-3p* relative expression by RT–qPCR of HepG2 cells transfected with siRNA for OGT and treated with 2-DG 5 mM for 48 h (lower panel). The lower panel shows the effective OGT protein reduction by siRNA treatment by western blot analysis (**b**). *PrimiR-483* (left *y* axis) and *miR-483-3p* (right *y* axis) relative expression by RT–qPCR, of HepG2 cells treated with azaserine 100 μM for 24 h. (**c**) Chromatin immunoprecipitation (ChIP) analysis for USF1 occupancy at the promoter of the *miR-483* (6841F-7100R) and at the 3′ UTR of UBE3A (3UTR-UBE3A) as control, by RT–qPCR (normalized on input DNA) in OGT-depleted HepG2 cells for 19 h. The BUB1 antibody was used as negative control. (**d**) (Left) Electrophoretic mobility shift assay (EMSA) analysis of nuclear extract (NE) from HepG2 cells transfected with siRNA for OGT or treated with azaserine 200 μM for 24 h using the *miR-483* E-box probe. The specific complexes are indicated with black arrows (b, c). EMSA for lanes siCTRL and siOGT was performed once. (Right) OGT, β-catenin, USF1 protein relative expression and glycosylation status (using RL2 antibody) of the nuclear extract by western blot from HepG2 cells transfected with siRNA for OGT or treated with azaserine 200 μM for 24 h. Densitometry analysis is shown. For statistical analysis, Student’s *t*-test was applied (**P<0.01; ***P<0.001).

OGT contributes to β-catenin stabilization^13, 14^ and is related to the insulin pathway;^15^ thus we investigated its role in the glucose-dependent regulation of *miR-483-3p*. We evaluated *miR-483-3p* expression following either OGT depletion or 2-DG treatment in HepG2 cells. Both OGT silencing by siRNA and 2-DG treatment led to the suppression of *miR-483-3p* expression, with the greatest effect achieved by the concomitant administration of 2-DG and siRNA for OGT (siOGT) (Figure 3a).

To confirm the regulation of *miR-483-3p* by OGT, we treated HepG2 cells with azaserine, a OGT inhibitor. As shown in Figure 3b, the compound reduced *miR-483-3p* and *primiR-483-3p* expression, suggesting an effect on its transcription.

To study if OGT modulates *miR-483-3p* expression by acting on the CTNNB1/USF1 complex, we conducted a chromatin immunoprecipitation analysis for USF1 occupancy at the promoter of *miR-483* (6841F-7100R) in HepG2 cells transfected with either siCTRL or siOGT. The 3′ UTR of the gene *UBE3A* (3UTR-UBE3A) was added as control. The siOGT treatment reduced the affinity between USF1 and the miR-483 E-box sequence, suggesting that OGT is fundamental for USF1 activity and in turn the regulation of the *miR-483-3p* expression (Figure 3c). BUB1 antibody was used as negative immunoprecipitation control, sonication and transfection controls are reported in Supplementary Figure S1A.

To confirm these data, we performed electrophoretic mobility shift assay analysis of HepG2 nuclear protein extracts obtained after transfection with OGT-siRNA or azaserine treatment; the *miR-483* E-box was used as probe. This showed that OGT inhibition reduces the specific binding of the USF1 complex to the *miR-483* E-box (Figure 3d, left). Western blot analysis of the nuclear protein extract confirmed OGT reduction after siOGT treatment. Despite the physiologically increased expression of OGT protein after azaserine treatment, a reduction of the overall glycosylation status was observed by using the O-GlcNAc antibody (RL2), which binds O-linked *N*-acetylglucosamine residues (Figure 3d, right). The effects of azaserine and of OGT-siRNA on *miR-483-3p* and *primiR-483* are reported in Supplementary Figure S1B. Based on our previously published evidence of an interplay between *miR-483-3p* and the TP53 pathway,^4^ we measured the mRNA expression of the TP53 transcriptional target *BBC3* gene upon silencing of OGT and 2-DG treatment. Decreased *miR-483-3p* expression was concomitant to increased *BBC3* RNA levels, index of TP53 activity (Supplementary Figure S1C).

Then, given that the *OGT* gene is a predicted target of the *miR-483-3p* by TargetScan (http://www.targetscan.org/vert_71/), we tested by western blot OGT expression in HepG2 cells transfected with either miR-483-3p mimic or anti-miR-483-3p (AMO-483-3p). Compared with the controls, no differences in OGT expression were observed (Supplementary Figure S1D), indicating that OGT is not a target of *miR-483-3p* in HepG2 cells.

To verify whether CTNNB1 directly interacts with OGT in our cell model, we performed an immunoprecipitation analysis in HepG2 cells transfected with a vector carrying USF1. We observed an interaction between CTNNB1 and OGT, suggesting that OGT regulated *miR-483-3p* expression by affecting the stability of CTNNB1 (Supplementary Figure S2).

### Effect of 2-DG on HepG2 cells *in vitro* and *in vivo*

Given that *miR-483-3p* was proven to have an oncogenic role in HCC^5^ and to inhibit the pro-apoptotic pathway induced by *miR-145-5p* and TP53,^4^ we hypothesize that 2-DG could reduce *miR-483-3p* expression and increase the efficacy of chemotherapeutic treatments in HCC cells.

**Figure 4:**
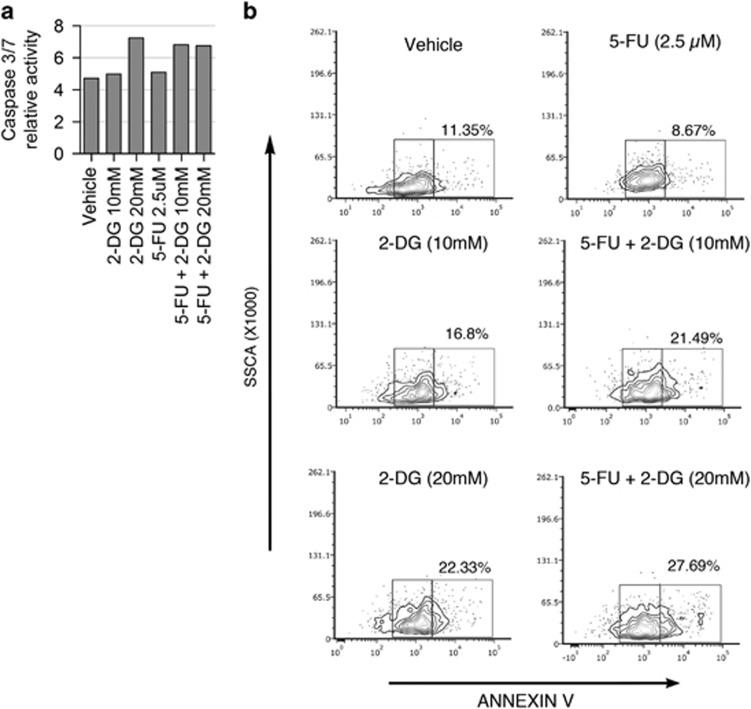
Effect of 2-DG and 5-FU combination *in vitro*. (**a**) Caspase 3/7 activation, normalized on ACTB protein expression measured by western blot (data not shown), and (**b**) Annexin V analysis of HepG2 cells treated with 2-DG (10 and 20 mM), 5-FU (2.5 μM) or in combination in Dulbecco’s modified Eagle’s medium (DMEM) low glucose for 24 h. Only bright annexin was measured as high apoptosis rate was detectable in treated cells.

Anti-*miR-483-3p* in combination with 2-DG was tested on HepG2 cells treated with different anticancer drugs, including sorafenib, doxorubicin and 5-FU. Inhibition of *miR-483-3p* significantly increased 5-FU-induced apoptosis in 2-DG treated cells (Supplementary Figure S3A). Next, we treated HepG2 cells with 2-DG (10 and 20 mM) alone or in combination with 5-FU (2.5 μM) and we evaluated caspase 3/7 activity. The combination of the two compounds resulted in higher caspase activity compared with the control (Figure 4a), which was confirmed by analysis of annexin V (Figure 4b). Reduction of the *miR-483-3p* expression is documented in Supplementary Figure S3B.

**Figure 5:**
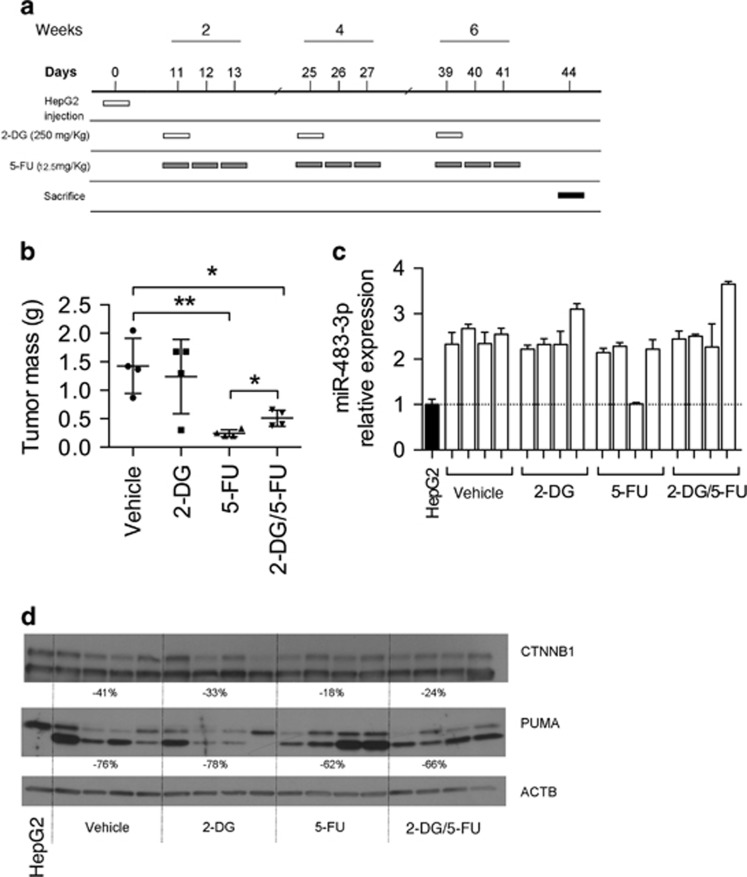
Effect of 2-DG and 5-FU combination *in vivo*. (**a**) Time table of the *in vivo* experiment. Sixteen NSG mice were randomly divided in four groups of treatment: vehicle (PBS), 2-DG, 5-FU and 2-DG+5-FU. Weeks and days of the different treatments are reported. At day 1, the mice were injected with 10 × 10^6^ HepG2 cells. At the second, fourth and sixth week, the mice were treated with 2-DG, 5-FU, both the drugs or the vehicle. Every day of treatment, the mice received a single intraperitoneal injection of 200 μl. Every drug or combination was dissolved in PBS. In (**b**), the total weight of xenografts obtained by NOD scid gamma (NSG) mice is reported. (**c**) *miR-483-3p* relative expression, normalized on RNU44, by RT–qPCR from the same samples (white bars) and from HepG2 cells before inoculation in mice (black bar). The graphs represents the means of technical triplicates with the respective s.d. For statistical analysis, Student’s *t*-test was applied (*P<0.05; **P<0.01). (**d**) Protein relative expression by western blot of CTNNB1 and PUMA in tumors induced in xenografts model. Densitometry analysis is shown. Every value is normalized on relative ACTB, and it reports the percentage of difference for every group of treatment vs the control group.

To evaluate the effect of the drugs *in vivo*, we generated a xenograft model in which NOD scid gamma mice were intraperitoneally inoculated with HepG2. Mice were treated with intraperitoneal injection of 2-DG (250 mg/kg one injection), or 5-FU (12.5 mg/kg for 3 consecutive days), or combination of the two drugs, or phosphate-buffered saline (PBS). We alternated a week of treatment with a week of rest, for a total of three cycles (Figure 5a). After 3 days from the last treatment, the mice were sacrificed and the xenografts were weighted. We observed reduction of tumor volume after treatment with 5-FU; however, addition of 2-DG did not improve 5-FU efficacy (Figure 5b). To explain this phenomenon, we analyzed *miR-483-3p* RNA expression together with the protein levels of PUMA and CTNNB1, which are targets of the miR. Compared with HepG2 cells, in almost all xenografts *miR-483-3p* showed significantly increased expression, concomitant with downregulation of its targets (Figure 5c) suggesting that the 2-DG, at the evaluated concentration and mode of administration, did not affect the expression of the miR *in vivo*.

## Discussion

In liver cancer, the expression of the *miR-483-3p* is related to the mutational status of *TP53* and *CTNNB1* genes and to impaired epigenetic mechanisms affecting the *IGF2/miR-483/H19* locus. The overexpression of of the *miR-483-3p* directly inhibits protein expression of the pro-apoptotic factor TP53 upregulated modulator of apoptosis (PUMA).^3, 4, 5^

Here we demonstrate that cellular glucose availability is involved in the modulation of *miR-483-3p* and identified OGT activity as a molecular link between glucose and *miR-483-3p* expression. OGT mediates the addition of a *N*-acetylglucosamine in O-glycosidic linkage (O-GlcNAcylation) to serine or threonine residues^16, 17^ and influences several cellular processes, such as epigenetic control of RNA transcription,^18^ insulin signaling,^15^ Wnt/β-catenin signaling^13, 14^ and mouse embryonic development.^19^ Inhibition of OGT activity results in decreased *miR-483-3p* expression, concurrently with weakened affinity of the transcriptional complex CTNNB1/USF1 to the responsive sequence upstream the *miR-483* gene. Although our data show that OGT influences the CTNNB1/USF1 complex acting at the responsive E-box element, we cannot exclude involvement of other molecular mechanisms. Indeed OGT is implicated in RNA transcription by O-GlcNAcylation of RNA polymerase II^20^ and of several transcription factors, such as SP1^21^ and MYC,^22^ in addition to CTNNB1.^13, 14^

The reprogramming of glucose metabolism and its epigenetics aspects are relevant to the development of new approaches to cancer treatment.^23, 24, 25^ In this regard, the antitumoral effects of 2-DG are under intense study,^26, 27^ and we found that 2-DG treatment inhibits *miR-483-3p* expression in HepG2 cells, probably by reducing the OGT substrate, and equally enhances the apoptotic rate of HepG2 cells treated with 5-FU. We decided to investigate the effects of co-treatment with 2-DG and 5-FU on HepG2 xenografts *in vivo*. By analyzing the engrafted tumor masses, we found that tumor load decreased less in the group of mice co-treated with 2-DG and 5-FU compared with the group treated with 5-FU alone. This could be explained by the fact that *miR-483-3p* was not affected by 2-DG at the dose and modality of administration used. Instead of the predicted reduction of *miR-483-3p* expression, the xenografts showed induction of the miRNA after 2-DG and 5-FU co-treatment. This could be due to a cellular selection of clones with higher expression of the miRNA, an event that we previously observed also studying TP53 activation.^4^

Altogether these results prove that the glucose/OGT/*miR-483-3p* signaling axis has an important role in the anti-apoptotic mechanisms of liver cancer and can be an important dowel to understand the oncogenic mechanisms that link metabolism to resistance to drugs and apoptosis. Therefore, *miR-483-3p* may be a promising target in the management of liver cancer patients, but the modality of its modulation should not include 2-DG or other drugs that cause general metabolic inhibition, an effect that could be counterproductive, as it would increase the selective pressure for the most adaptive cancer cells.

## Materials and methods

### Cell lines, drugs treatment and transfection

HepG2 cell line, from American Type Culture Collection (ATCC, Manassas, VA, USA), were cultured with Dulbecco’s modified Eagle’s medium, with low or high glucose concentration (1 g and 4.5 g per liter, respectively), completed with 10% fetal bovine serum European approved, l-glutamine, penicillin, streptomycin and Normocin (100 ug/ml) (InvivoGen, San Diego, CA, USA). The 2-DG (D6134), 5-FU (F6627) and azaserine (A4142) were obtained from Sigma Aldrich (St Louis, MO, USA). *MiR-483-3p* precursor and negative control 2 ribo-oligonucleotide (NC2) were from Ambion (Thermo Fisher Scientific, Waltham, MA, USA). Anti-miRNA oligonucleotides (AMO) against *miR-483-3p* and against the GFP gene (AMO negative control) were from Fidelity System (Gaithersburg, MD, USA). RNA interfering for *OGT* (sc-40780) and scramble control (sc-37007) were from Santa Cruz Biotechnology (Santa Cruz, CA, USA). Transfection of miRNAs, AMOs and expression vectors was carried out with Lipofectamine 2000 (Thermo Fisher Scientific, Walmar, MA, USA) in accordance with the procedures of the manufacturer.

### RNA extraction, cDNA and quantitative real-time PCR (RT–qPCR) for miRNA and mRNA

Purification of total RNA using Qiazol reagent (Qiagen, Hilden, Germany) was carried out following the manufacturer’s instructions. To avoid genomic contamination in the quantification of pre-mRNA and pri-miRNA, RNA samples used for the analysis were previously treated with DNase (TURBO DNA-free Kit; Thermo Fisher Scientific). To analyze the mature miRNA expression normalized on *RNU44*, we synthesized specific miRNA complementary DNA (cDNA) from RNA samples. In all, 25 ng of total RNA were reverse transcribed using the cDNA Reverse Transcription Kit (Thermo Fisher Scientific) and the specific stem loop RT primers designed with a modification to include the UPL #21 complementary sequence.^28, 29^ The mix for the reverse transcription reaction included: 5 μl of RNA (5 ng/μl); 2 μl of 10X reverse transcription buffer; 0.2 μl of 100 mM dNTPs; 0.2 μl of RNase inhibitor (20 U/μl); 1 μl of MultiScribe reverse transcriptase (50 U/μl); 0.5 μl of specific loop primers (1 μM) (diluted in TRIS/HCl 2.5 mM pH 8.5) for miRNA and RNU44; and nuclease-free water to reach the final volume of 20 μl. The pulsed RT reaction was carried out in 0.2 ml tubes at 16 °C for 30 min, followed by 60 cycles (30 °C for 30 s, 42 °C for 30 s, 50 °C for 1 s) with the final step of 85 °C for 5 min, using a thermal cycler GeneAmp PCR System 9700 (Thermo Fisher Scientific). Then qPCRs were performed. In total, 10 μl of qPCR reaction included: 3.4 μl of RNase-free water; 5 μl of Mastermix 2X (FastStart Universal Probe Mastermix, Roche, Basilea, Svizzera); 0.5 μl of specific miRNA primer forward (20 μM); 0.5 μl of universal reverse primer (20 μM); 0,1 μl of Universal Probe Library 21 (4686942001, Roche); 1 μl of cDNA diluted.

To quantify mRNAs normalized on *ACTB* expression, cDNA was generated using the High Capacity cDNA reverse transcription kit (Thermo Fisher Scientific). The 20 μl of reverse transcription reaction included: 8.2 μl nuclease-free water; 2 μl of 10X reverse transcription buffer; 0.8 μl of 100 mM dNTPs; 1 μl of RNase inhibitor (20 U/μl); 1μl of MultiScribe reverse; 2 μl of random primers; 1 μl transcriptase (50 U/μl); and 5 μl of RNA (100 ng/μl). The reactions were carried out in 0.2 ml tubes at 25 °C for 10 min, 37 °C for 2 h and 85 °C for 5 min, using the thermal cycler GeneAmp PCR System 9700 (Thermo Fisher Scientific). The cDNA obtained was diluted 1:2 and used for the qPCR using the kit FastStart Universal Probe Mastermix. The 10 μl of reaction included: 2.4 μl of RNase-free water, 5 μl of Mastermix 2X, 0.5 μl of specific mRNA primer forward (20 μM), 0.5 μl of specific mRNA primer reverse; 0.1 μl of specific probe (Universal probe library system, Roche) and 2 μl of cDNA diluted. Primer sequences and relative fluorescent 6-Carboxyfluorescein probes were obtained by the ‘Universal Probe Library Assay Design Center’ online software (https://lifescience.roche.com). When the identification of a suitable UPL probe was not possible, SYBR green technologies were used for the quantification of a specific sequence (QuantiFast SYBR Green PCR Kit; Qiagen), in this case the 10 μl of reaction included: 2.8 μl of RNase-free water, 5 μl of SYBR green Mastermix 2X (Qiagen); 0.1 μl of specific forward and reverse primer (20 μM); and 2 μl of cDNA diluted (1:2).

qPCR reactions were conducted using an ABI PRISM 7900HT Sequence Detection System (Thermo Fisher Scientific) or the CFX96 Touch Real-Time PCR Detection System (Bio-Rad, Hercules, CA, USA). All samples were run in triplicate, and the levels of miRNA and mRNA were measured using the threshold cycle (Ct). The amount of target, normalized to an endogenous reference (*RNU44* for miRNA and *ACTB* for mRNA), is given by 2^−ΔCt^, with formula 2–(Ct sample–(mean triplicate of the Ct reference)) in base to the comparative Ct method (Applied Biosystems User Bulletin no. 2).

Primer sequences used are provided in Supplementary Table 1.

### Protein extraction, quantification and western blot

Total protein extraction were performed by using M-PER Mammalian Protein Extraction Reagent (Thermo Fisher Scientific) freshly supplemented with protease and phosphatase inhibitors (Thermo Scientific); whereas the NePer kit (Thermo Fisher Scientific) was used for nuclear and cytoplasmic extraction following the manufacturer’s instructions. Protein concentration was evaluated by spectrophotometric analysis using the Bio-Rad Protein Assay Dye Reagent (Bio-Rad) and the GeneQuant pro spectrophotometer (Biochrom Ltd., Cambrige, UK). Up to 50 μg of protein were loaded onto gel Criterion TGX Stain Free Gel, 4–20% (Bio-Rad) and run at 80 V for 2 h. Proteins were transferred in polyvinylidene difluoride membrane at 20 V overnight. Blots were blocked with non-fat dry milk 5% (Cell Signaling Technology, Danvers, MA, USA) for 1 h at room temperature and incubated with specific primary antibody from 2 h to overnight at 4 °C. After Tris-buffered saline-Tween washes, blots were incubated with peroxidase-conjugated anti-mouse or anti-rabbit antibodies for 1 h at room temperature. Detection was conducted by chemiluminescent assay (Pierce ECL western blotting substrate, Thermo Fisher Scientific). To quantify western blot signals, digital images of autoradiographs were acquired, and band signals were quantified in the linear range of the scanner using the software ImageJ (http://imagej.net/Welcome). Protein loading were evaluated by measuring the β-actin or vinculin protein expression. The list of the antibodies used is provided in Supplementary Table 2.

### Luciferase assay

HepG2 cells were seeded in a 96-well plate and transfected with the DNA vectors pRLTK (Promega, Madison, WI, USA), which expresses the reference renilla luciferase, and the PGL4 Luciferase Reporter Vector, (Promega) containing the E-Box sequence interacting with the CTNNB1/USF1 complex (pGL4 6839-6910), expressing the luciferase. The DNA vectors were transfected by Lipofectamine (Thermo Fisher Scientific) following the manufacturer’s procedure. After 48 h, the luminescence was evaluated with the Dual-Glo Luciferase Assay System (Promega) using a Lumat LB 9507 the luminometer (Berthold Technologies, Bad Wildbad, Germany).

### Electrophoretic mobility shift assay

Protein/DNA binding was determined by electrophoretic mobility shift assay according to the manufacturer’s procedure (LightShift Chemiluminescent EMSA Kit; Pierce, Thermo Fisher Scientific) with biotinylated probes (Fidelity System, Gaithersburg, MD, USA; Supplementary Table 1). Nuclear protein extract (NE) from HepG2 were incubated with *miR-483* E-box wild-type biotinylated probe (OligoBio) with or without 100 × E-box unlabeled probe, and/or with specific polyclonal USF1 antibody. The DNA–protein complexes were resolved on 5% nondenaturing acrylamide gels and visualized by chemoluminescent assay and exposure to autoradiographic films.

### Immunoprecipitations (chromatin immunoprecipitation)

Immunoprecipitation was executed using the kit Magna ChIP Protein A+G Magnetic Beads (Merck Millipore, Billerica, MA, USA) following the manufacturer’s instructions. Cells were fixed with formaldehyde (1% 10 min at room temperature), glycine was added to stop the reaction and after 5 min, then washed with ice-cold cold PBS to remove completely the formaldehyde. Cells were collected by scraping in PBS ice cold containing 1x proteinase inhibitor, pelleted and lysed with buffer containing proteinase inhibitors. After vortex and centrifugation, the supernatant was separated and the nuclear lysis buffer added to the pellet. After centrifugation to remove the debris, the protein nuclear extract was sonicated using an IKA Labor technik U200 S Control apparatus (IKA-Works, Staufen im Breisgau, Germany, 30% amplitude, 0.3-s pulse rate or 30% cycle duty for 1 min). Part of the lysate sonicated was denatured and run for gel electrophoresis to confirm that the length of the DNA fragments was between the 200 and 1000 base pairs. The rest of the lysate was centrifuged to remove debris and used for the different immunoprecipitations. Each immunoprecipitation was conducted with 50 μl of lysate diluted in 450 μl of dilution buffer containing protease inhibitors. In all, 5 μl were removed and keep at −80 °C (input). For each experimental condition, 1 μg of USF1 antibody or controls (mouse IgG, BUB1 antibody) and 20 μl of fully resuspended protein A/G magnetics beads were added to the diluted lysate. All the reactions were incubated overnight at 4 °C with rotation. The protein A/G magnetic beads were pellet with the magnetic separator and washed with specific salt solutions. Then elution buffer containing the proteinase K was added and the samples were incubated at 62 °C for 2 h and 95 °C for 10 min with shaking. Beads were separated with the magnetic separator and the supernatant containing the DNA associated at the protein complexes were purified by columns. In all, 2 μl of the obtained DNA were used for qPCR to quantify the *miR-483-3p* promoter region using the primers 6841_F and 7100_R, or the 3’UTR region of UBE3A as control. For qPCRs, every sample was analyzed in triplicate.

### Caspase 3/7 activity and Annexin V flow cytometric analysis

HepG2 and Hep3B cells were seeded in a 96-well plate with opaque borders. After the transfection and/or the drug treatment, the cellular apoptosis was evaluated by the Caspase-Glo 3/7 Assay (Promega), following the manufacturer’s procedure. The luminescence was evaluated by the Veritas Microplate Luminometer (Turner BioSystems, Promega). Every sample was analyzed in triplicate.

Another assay to measure apoptosis was performed by fluorescence-activated cell sorter using Annexin V-APC conjugated detection kit (Enzo Life Sciences, Farmingdale, NY, USA). After washing in PBS, cells were stained using Annexin V antibody in specific binding buffer for 10 min in darkness, according with the manufacturer’s procedure. Then cellular apoptosis was analyzed by flow cytometer (FACSCalibur, BD).

### Mice treatment and management

Animal studies were approved by institutional and government ethical committees (823/2015-PR). Sixteen NOD scid gamma (Charles River Breeding Laboratories, Wilmington, MA, USA) male mice were used (four for each group). No mice randomization was performed and no blinding procedures were performed. All mice were housed in the Aging Research Center (CeSI-MeT, Chieti, Italy) animal facility. They were maintained on a 12-h light/dark cycle with *ad libitum* access to water and normal chow. The suffering of the mice was evaluated using the ‘mouse grimace scale'^30^ by the responsible of the stabulary and no suffering status was registered during the management and the treatment. The cages, the water, the chow and every items or substance that was in contact with the mice were sterile. The mice of 6 weeks were inoculated with 10^7^ HepG2 cells by intraperitoneal injection using a 26-gauge syringe and divided in four groups of treatment: PBS, 2-DG, 5-FU and 2-DG+5-FU.

Then, three cycles of treatment were executed at 11, 25 and 39 day from the HepG2 injection. The administration of 2-DG (250 mg/kg) was once at treatment, whereas for 5-FU was decided to execute three separate administration during the week of treatment (12.5 mg/kg each). Each drug was dissolved in sterile PBS, and administered by intraperitoneal injection at a final volume of 200 μl for mouse. We used the no toxic dose of 2-DG^31^ alone or in combination with 5-FU to treat mice. At 45 days from the injection of the cells, mice were sacrificed by CO_2_ chamber. Tumor masses were photographed, weighted and putted in liquid nitrogen until RNA and protein extraction.

### Statistical analysis

All the *in vitro* experiments were replicated either twice or in similar conditions or in different cellular models unless not specified in the legends. The variation within each group has been evaluated as s.d. and for the *in vivo* results, the Levene's test was not significant to indicate the homoscedasticity of the data. Significance was determined with the two-tailed Student’s *t*-test. A *P*-value threshold <0.05 was considered significant (marked as *), *P* value<0.01, <0.001 and <0.0001 are marked as **, *** and ****, respectively. In graphs, values were presented as the mean±s.d.

## References

[bib1] Ferlay J, Shin HR, Bray F, Forman D, Mathers C, Parkin DM. Estimates of worldwide burden of cancer in 2008: GLOBOCAN 2008. Int J Cancer 2010; 127: 2893–2917. 21351269 10.1002/ijc.25516

[bib2] Lozano R, Naghavi M, Foreman K, Lim S, Shibuya K, Aboyans V et al. Global and regional mortality from 235 causes of death for 20 age groups in 1990 and 2010: a systematic analysis for the Global Burden of Disease Study 2010. Lancet 2012; 380: 2095–2128. 23245604 10.1016/S0140-6736(12)61728-0 PMC10790329

[bib3] Veronese A, Visone R, Consiglio J, Acunzo M, Lupini L, Kim T et al. Mutated beta-catenin evades a microRNA-dependent regulatory loop. Proc Natl Acad Sci USA 2011; 108: 4840–4845. 21383185 10.1073/pnas.1101734108 PMC3064338

[bib4] Lupini L, Pepe F, Ferracin M, Braconi C, Callegari E, Pagotto S et al. Over-expression of the miR-483-3p overcomes the miR-145/TP53 pro-apoptotic loop in hepatocellular carcinoma. Oncotarget 2016; 7: 31361–31371. 27120784 10.18632/oncotarget.8913 PMC5058762

[bib5] Veronese A, Lupini L, Consiglio J, Visone R, Ferracin M, Fornari F et al. Oncogenic role of miR-483-3p at the IGF2/483 locus. Cancer Res 2010; 70: 3140–3149. 20388800 10.1158/0008-5472.CAN-09-4456 PMC4303586

[bib6] Kahn A. Transcriptional regulation by glucose in the liver. Biochimie 1997; 79: 113–118. 9209706 10.1016/s0300-9084(97)81501-5

[bib7] Corre S, Galibert MD. Upstream stimulating factors: highly versatile stress-responsive transcription factors. Pigment Cell Res 2005; 18: 337–348. 16162174 10.1111/j.1600-0749.2005.00262.x

[bib8] Cognard E, Dargaville CG, Hay DL, Shepherd PR. Identification of a pathway by which glucose regulates beta-catenin signalling via the cAMP/protein kinase A pathway in beta-cell models. Biochem J 2013; 449: 803–811. 23198873 10.1042/BJ20121454

[bib9] Dynkevich Y, Rother KI, Whitford I, Qureshi S, Galiveeti S, Szulc AL et al. Tumors, IGF-2, and hypoglycemia: insights from the clinic, the laboratory, and the historical archive. Endocr Rev 2013; 34: 798–826. 23671155 10.1210/er.2012-1033

[bib10] Livingstone C. IGF2 and cancer. Endocr Relat Cancer 2013; 20: R321–R339. 24080445 10.1530/ERC-13-0231

[bib11] Onodera Y, Nam JM, Bissell MJ. Increased sugar uptake promotes oncogenesis via EPAC/RAP1 and O-GlcNAc pathways. J Clin Invest 2014; 124: 367–384. 24316969 10.1172/JCI63146 PMC3871217

[bib12] Butkinaree C, Park K, Hart GW. O-linked beta-*N*-acetylglucosamine (O-GlcNAc): extensive crosstalk with phosphorylation to regulate signaling and transcription in response to nutrients and stress. Biochim Biophys Acta 2010; 1800: 96–106. 19647786 10.1016/j.bbagen.2009.07.018 PMC2815129

[bib13] Olivier-Van Stichelen S, Guinez C, Mir AM, Perez-Cervera Y, Liu C, Michalski JC et al. The hexosamine biosynthetic pathway and O-GlcNAcylation drive the expression of beta-catenin and cell proliferation. Am J Physiol Endocrinol Metab 2012; 302: E417–E424. 22114026 10.1152/ajpendo.00390.2011

[bib14] Olivier-Van Stichelen S, Dehennaut V, Buzy A, Zachayus JL, Guinez C, Mir AM et al. O-GlcNAcylation stabilizes beta-catenin through direct competition with phosphorylation at threonine 41. FASEB J 2014; 28: 3325–3338. 24744147 10.1096/fj.13-243535 PMC4101651

[bib15] Yang X, Ongusaha PP, Miles PD, Havstad JC, Zhang F, So WV et al. Phosphoinositide signalling links O-GlcNAc transferase to insulin resistance. Nature 2008; 451: 964–969. 18288188 10.1038/nature06668

[bib16] Slawson C, Housley MP, Hart GW. O-GlcNAc cycling: how a single sugar post-translational modification is changing the way we think about signaling networks. J Cell Biochem 2006; 97: 71–83. 16237703 10.1002/jcb.20676

[bib17] Hart GW, Housley MP, Slawson C. Cycling of O-linked beta-*N*-acetylglucosamine on nucleocytoplasmic proteins. Nature 2007; 446: 1017–1022. 17460662 10.1038/nature05815

[bib18] Chen Q, Chen Y, Bian C, Fujiki R, Yu X. TET2 promotes histone O-GlcNAcylation during gene transcription. Nature 2013; 493: 561–564. 23222540 10.1038/nature11742 PMC3684361

[bib19] Shafi R, Iyer SP, Ellies LG, O'Donnell N, Marek KW, Chui D et al. The O-GlcNAc transferase gene resides on the X chromosome and is essential for embryonic stem cell viability and mouse ontogeny. Proc Natl Acad Sci USA 2000; 97: 5735–5739. 10801981 10.1073/pnas.100471497 PMC18502

[bib20] Comer FI, Hart GW. Reciprocity between O-GlcNAc and O-phosphate on the carboxyl terminal domain of RNA polymerase II. Biochemistry 2001; 40: 7845–7852. 11425311 10.1021/bi0027480

[bib21] Majumdar G, Harmon A, Candelaria R, Martinez-Hernandez A, Raghow R, Solomon SS. O-glycosylation of Sp1 and transcriptional regulation of the calmodulin gene by insulin and glucagon. Am J Physiol Endocrinol Metab 2003; 285: E584–E591. 12900380 10.1152/ajpendo.00140.2003

[bib22] Buren S, Gomes AL, Teijeiro A, Fawal MA, Yilmaz M, Tummala KS et al. Regulation of OGT by URI in response to glucose confers c-MYC-dependent survival mechanisms. Cancer Cell 2016; 30: 290–307. 27505673 10.1016/j.ccell.2016.06.023

[bib23] Hay N. Reprogramming glucose metabolism in cancer: can it be exploited for cancer therapy? Nat Rev Cancer 2016; 16: 635–649. 27634447 10.1038/nrc.2016.77 PMC5516800

[bib24] Shang RZ, Qu SB, Wang DS. Reprogramming of glucose metabolism in hepatocellular carcinoma: progress and prospects. World J Gastroenterol 2016; 22: 9933–9943. 28018100 10.3748/wjg.v22.i45.9933 PMC5143760

[bib25] Wong CC, Qian Y, Yu J. Interplay between epigenetics and metabolism in oncogenesis: mechanisms and therapeutic approaches. Oncogene (e-pub ahead of print 16 January 2017; doi:10.1038/onc.2016.485). 10.1038/onc.2016.485 PMC5485177 28092669

[bib26] Stein M, Lin H, Jeyamohan C, Dvorzhinski D, Gounder M, Bray K et al. Targeting tumor metabolism with 2-deoxyglucose in patients with castrate-resistant prostate cancer and advanced malignancies. Prostate 2010; 70: 1388–1394. 20687211 10.1002/pros.21172 PMC4142700

[bib27] Raez LE, Papadopoulos K, Ricart AD, Chiorean EG, Dipaola RS, Stein MN et al. A phase I dose-escalation trial of 2-deoxy-D-glucose alone or combined with docetaxel in patients with advanced solid tumors. Cancer Chemother Pharmacol 2013; 71: 523–530. 23228990 10.1007/s00280-012-2045-1

[bib28] Chen C, Ridzon DA, Broomer AJ, Zhou Z, Lee DH, Nguyen JT et al. Real-time quantification of microRNAs by stem-loop RT-PCR. Nucleic Acids Res 2005; 33: e179. 16314309 10.1093/nar/gni178 PMC1292995

[bib29] Varkonyi-Gasic E, Wu R, Wood M, Walton EF, Hellens RP. Protocol: a highly sensitive RT-PCR method for detection and quantification of microRNAs. Plant Methods 2007; 3: 12. 17931426 10.1186/1746-4811-3-12 PMC2225395

[bib30] Langford DJ, Bailey AL, Chanda ML, Clarke SE, Drummond TE, Echols S et al. Coding of facial expressions of pain in the laboratory mouse. Nat Methods 2010; 7: 447–449. 20453868 10.1038/nmeth.1455

[bib31] Huang CC, Wang SY, Lin LL, Wang PW, Chen TY, Hsu WM et al. Glycolytic inhibitor 2-deoxyglucose simultaneously targets cancer and endothelial cells to suppress neuroblastoma growth in mice. Dis Models Mech 2015; 8: 1247–1254. 10.1242/dmm.021667 PMC4610240 26398947

