# Supplementary material for: Regulation of miR-483-3p by the O-linked N-acetylglucosamine transferase links chemosensitivity to glucose metabolism in liver cancer cells
Source: Oncogenesis. 2017 May 8;6(5):e328–. doi: 10.1038/oncsis.2017.35 (PMC5523068; doi:10.1038/oncsis.2017.35)
Supplement: Supplementary Material [file oncsis201735x1.pdf]

Supplementary table and figures

Table S1: Primer sequences.

Primer name	*Primer sequence*
RT_483-3p	GTTGGCTCTGGTGCAGGGTCCGAGGTATTCGCACCAGAGCCAACAAGACG
RT_U44	GTTGGCTCTGGTGCAGGGTCCGAGGTATTCGCACCAGAGCCAACAGTCAGTT
miR-483-3p forward	GCGGCGGTCACTCCTCTCCTC
RNU44 forward	GCGGCGGCCTGGATGATGATAG
Universal reverse	GTGCAGGGTCCGAGGT
U51_primiR-483_F	AGAAGGGAGTGGTTCCATCA
U51_primiR-483_R	AGCTGCTGAGACAGGGAGAC
U40_IGF2_forward	ACACCCTCCAGTTCGTCTGT
U40_IGF2_reverse	GAAACAGCACTCCTCAACGA
483_Ebox wt	ACCTGACACTCACCACGTGACATCTTTACCACC
483P_6841_Forward	CGCTCCTTACTGGCAGAGG
IGF2_7100_Reverse	TCCTCTCAGGAAAGCCTGGT
U15_UBE3A_F	CAGCTTACCTTGAGAACTCGAAA
U15_UBE3A_R	AAATCAATTCTAGCGCCTTTCTT

Table S2: Primary and secondary antibodies used for western blot and immunop recipitation experiments. In the column Experiment, **"**W.B." indicates western blot while "Ip" is for immunoprecipitation experiments.

Antibody	*Brand*	*Code*	*Secondary*	*Experiment*
CTNNB1 (D-10)	Santa Cruz	sc-133239	Mouse	WB
BUB1 (B3)	Santa Cruz	sc.356685	Mouse	IP
O-GlcNAc (RL2)	Santa Cruz	sc-59624	Mouse	WB
O-GlcNAc transferase (H-300)	Santa Cruz	sc-32921	Rabbit	WB/IP
USF1 (C-20)	Santa Cruz	sc-229	Rabbit	WB/IP
Vinculin (H-300)	Santa Cruz	sc-5573	Rabbit	WB
β-actin	Cell Signaling	4967	Rabbit	WB
Goat anti-mouse IgG-HRP	Santa Cruz	sc-2005		WB, IP
Goat Anti-Rabbit IgG (H + L)-HRP Conjugate	Biorad	170-6515		WB, IP

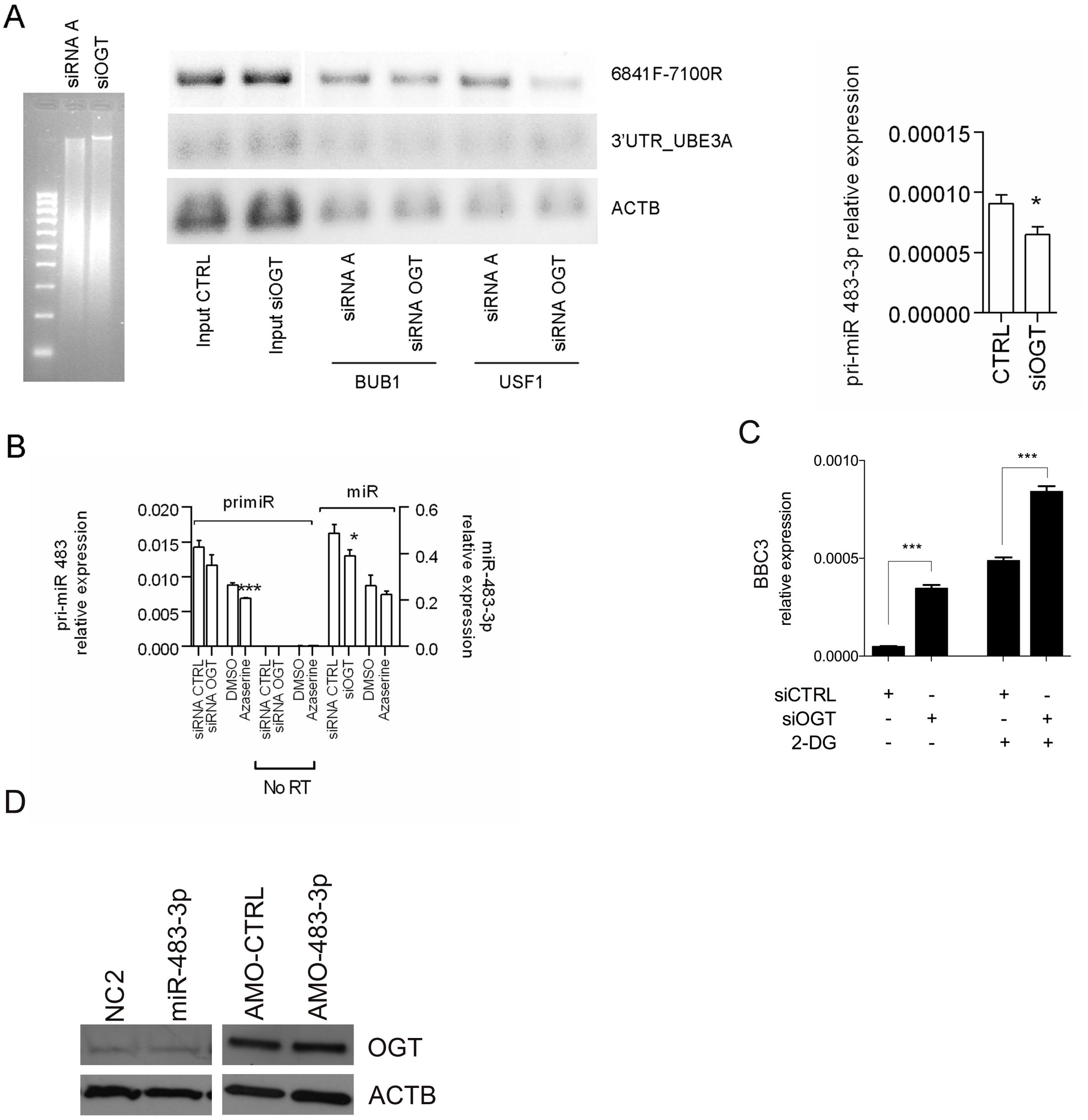

Supplementary Figure S1.

A. Controls of ChIP analysis reported in Figure 3C. Gel electrophoresis of input DNA fragment in 2% agar gel after sonication (left panel). Gel electrophoresis of the RT-qPCR products (central panel), and *primiR-483* relative expression by RT-qPCR (right panel). **B.** *PrimiR-483* and *miR-483-3p* relative expression of HepG2 cells transfected with siRNA for OGT or treated with azaserine 200 µM for 24 hours. For *primiR-483* expression is reported the No RT control. **C.** *BBC3* relative expression in HepG2 cells transfected with either the control (siCTRL) or the siRAN against *OGT* gene in two different conditions. The RT-qPCR was normalised on the *ACTB* expression. **D.** Western blot analysis using OGT antibody (AbOGT) in HepG2 cells transfected with the negative control 2 (NC2), the mimic miR-483-3p, the anti-miR negative control (AMO-CTRL) and the antimiR-483-3p (AMO-483-3p). HepG2 cells were harvest after 48 hours of transfection. Student's *t*-test was used to determine statistical significance of the differences between experimental groups. P value >0.05 was considered not significant, between 0.01 to 0.05 (*) significant, between 0.001 to 0.01 (**) very significant, and <0.001 (***) extremely significant.

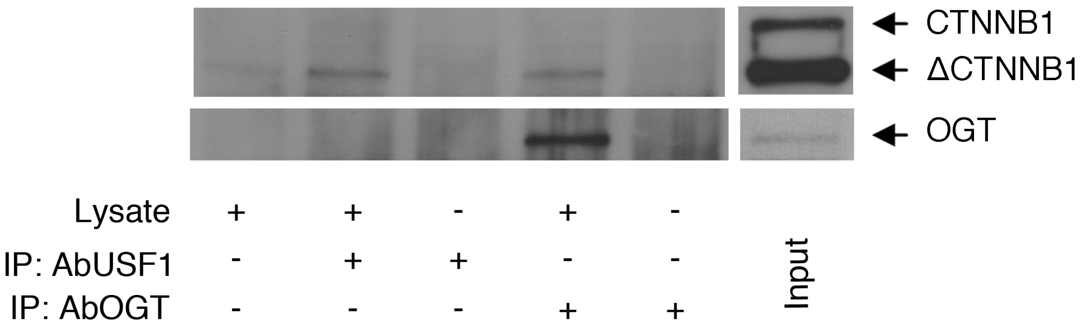

Supplementary Figure S2. Immunoprecipitation (IP) analysis using either USF1 or OGT antibodies (AbUSF1, AbOGT) in HepG2 cells transfected with a USF1 carrying vector. Specific bands are indicated with black arrows.

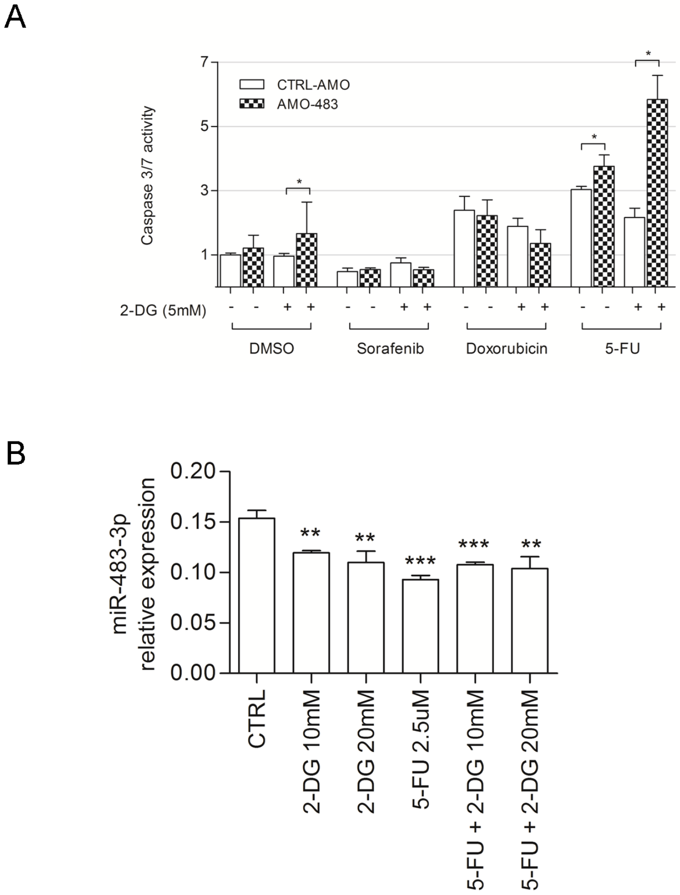

Supplementary Figure S3. A. Caspase 3/7 absolute activation of HepG2 cells transfected with oligonucleotide antimiR-483 (AMO-483) or control (CTRL-AMO) and treated with 2-DG 5 mM, sorafenib, doxorubicin and 5-FU 25 µM (single treatments or in combination with 2-DG) for 48 hours. All the values are normalized on control treated with the vehicle and transfected with the control antimiR. **B.** *miR-483-3p* relative expression in HepG2 cells treated with 2-DG 10 mM or 20 mM, or with 5-FU 2.5uM or in combination. These data are referred to the experiment reported in Figure 4A. Student's *t*-test was used to determine statistical significance of the differences between experimental groups. P value >0.05 was considered not significant, between 0.01 to 0.05 (*) significant, between 0.001 to 0.01 (**) very significant, and <0.001 (***) extremely significant.
